# Users’ participation and social influence during information spreading on Twitter

**DOI:** 10.1371/journal.pone.0183290

**Published:** 2017-09-13

**Authors:** Xin Zhang, Ding-Ding Han, Ruiqi Yang, Ziqiao Zhang

**Affiliations:** Shanghai Key Laboratory of Multi-dimensional Information Processing, School of Information Science and Technology, East China Normal University, Shanghai, People’s Republic of China; Beijing University of Posts and Telecommunications, CHINA

## Abstract

Online Social Networks generate a prodigious wealth of real-time information at an incessant rate. In this paper we study the empirical data that crawled from Twitter to describe the topology and information spreading dynamics of Online Social Networks. We propose a measurement with three measures to state the efforts of users on Twitter to get their information spreading, based on the unique mechanisms for information retransmission on Twitter. It is noticed that small fraction of users with special performance on participation can gain great influence, while most other users play a role as middleware during the information propagation. Thus a community analysis is performed and four categories of users are found with different kinds of participation that cause the information dissemination dynamics. These suggest that exiting topological measures alone may reflect little about the influence of individuals and provide new insights for information spreading.

## Introduction

In recent years, the impact of online social networks and their rapid growth have changed the patterns of human interaction and information dissemination among population in modern society. Twitter is a popular tool to quickly post and exchange text messages limited by 140 characters. In 2012, over 100 million users posted 340 million tweets on an average day [1, 2]. This platform represents a wide variety of communications, going from personal to those coming from traditional mass media, being suitable for conducting computational or social science analysis [3–5].

Most recent studies based on Twitter focused on the topology structures of user relationships [1, 6, 7], information trends [8], and community interactions [9–11]. Some authors conducted analysis to study the models of information spreading, prediction of social relations or collective attention, and the keys to propagating information on online social networks [12–18]. In studying these generative and analytical models, the microscopic dynamics has attracted many researchers, who focused on the patterns and characteristics of online human behaviors by empirical analysis [19–22].

However, all messages on Twitter may be identified using keywords called hashtags [23]. This mechanism generates the trending topics, and people use them to discuss and exchange ideas without the necessity of having any explicit relation. This means that the amount of messages that users can read is not related to the amount of the accounts that they subscribed. This also implies that the models based on cascades evolution or propagation path are not accurate and reasonable to explain the efforts of individuals to get their information spreading [24–27].

Meanwhile, the attraction or popularity of the content is an important factor that characterizes the influence of a node. According to different emphases of research task, the recent researches of information spreading can be summarized as the information-centric researches and user-centric researches [28]. In this work, we focused on user-centric researches and studied the users’ action and connection construction. Our main goal was to characterize the user efforts to influence more practically the information flow dynamics and growth of messages cascades on the Twitter network. We captured the data of outbreak and spread of an event that took place for a week in December 2015 on Twitter. This process was conducted with Twitter REST API. We also built two networks, the network where users followed each other and the network where information actually flowed, to represent and analyze the phenomena.

We proposed a measurement with three measures, user engine, user enthusiasm and user duration, to characterize users’ efforts in their participation of each conversation, in view of the retransmissions they gained due to such participation. The results indicated that users who gained large amount of retransmissions presented a large value of user engine and small values of user enthusiasm and user duration.

Furthermore, we studied the community structure of information flow network [29, 30] based on this pattern of participation, and found four categories of users with highly homogeneous profiles and participations that closely related to the information flow dynamics.

Section 2 describes the Twitter platform and the dataset we collected, and the two networks we built, and the special mechanisms for information propagation on Twitter. In Section 3, we focus on the empirical results of the measurements that lead us to state the users’ participation, influence and the information spreading process. The user categories and how they determine the information spreading are given, too.

## Methods

### Twitter and dataset

The first noticeable mechanism for users to communicate with each other on Twitter is that individuals can follow and be followed by other people. This generates the network of Twitter, a directed graph where users are connected with each other through explicit relation between them. Previous studies focused on the features and topology of this network, such as non-power-law follower distribution, short effective diameter, and low reciprocity [31]. Users interactions and modular structures are analyzed based on this network [32, 33].

Another important mechanism on Twitter is the retweet, where individuals deliver their own endorse of the information they read and make them visibility in the Twitter network by retweeting [34]. All messages no matter original or retweeted can be identified using keywords called hashtag, generating the trending topics. Users discuss and exchange ideas, read and retweet messages by hashtag, without the necessity of following others.

Twitter REST APIs [35] allow us to build our dataset. They provide programmatic access to read and write Twitter data. That means that the things obtained using Twitter APIs are all the data that Twitter is ready to make public. We can use them to create a new Tweet, read user profile and follower data, and more. So that our dataset complies with the terms of service for Twitter and there is no ethical problem for our study. The REST APIs identify Twitter applications and users using OAuth [36]; responses are in JSON format. Of course, there is a rate limit of Twitter REST APIs. The rate limit window is divided into 15 minute chunks per endpoint. We were limited to program with multiple threads, requesting 200 tweets per minute through the Twitter REST APIs to capture enough data.

We used the Search API [37] of Twitter REST APIs to get Twitter messages, with the query operator #AFCvMCFC to collect the messages containing the hashtag “AFCvMCFC”. The response [38] let us gain the message ID, user ID and all the detail information of each message. Based on the user ID and followers API [39] of Twitter REST APIs, we collected the information of users who participated in the activity and built *followers* *network*. Based on the detail information of each message, we then built *retweets* *network*.

### Information spreading mechanism on Twitter

On Twitter, the amount of users’ potential readers strongly depends on their in-degree of the *followers* *network*. This network, where nodes represent the users who participated in specific interaction and edges are created according to who follows who, is actually a partition of the global network. The *retweets* *network*, where nodes represent participants and edges are established according to who retransmits whose messages. These established edges are all directed and weighted based on the amount of retweeting actions.

The crawled data need some preprocessing. There may be missing and break point in the message spreading chain in building the *retweets* *network*. For example, User A posted a message, User B retweet from User A, then User C retweet from User B, but in the acquisition process we lost the data of whom User C retweeted from, then we could not figure out which one that User C retweeted from. Therefore, in the data preprocessing, a user was selected randomly as the missing user. Meanwhile, the data of profile and action were not complete for each user. Some data are lack of the status of retweet, some are lack of the time of retweet, while some others are lack of detail description or screen-name of the user. The study on the features of users’ action began with filtering the dataset and removing the data without profile or retweet action. The filtered data were used to study the statistical features. Obviously, this kind of preprocessing introduced some random factors that make the data points homogeneous to some extent. This will be discussed Section 3.

We considered *K*_*in*_/*K*_*out*_ to measure how popular a user is from the perspective of followers network, as the phenomenon of “rich-get-richer” agglomeration of popularity was found in many recent researches [40, 41]. *K*_*out*_ is the out-degree of a user in followers network. *K*_*in*_, the in-degree of a user in followers network, represents the amount of followers of the user. However, in social network, many users take the initiative to follow others to get followers. Some unpopular users can also gain large amount of followers. Therefore, one can’t judge whether a user is popular or not just in view of *K*_*in*_. On the other hand, *K*_*in*_/*K*_*out*_ represents on average how much attention a user can get when he/she follows others. *K*_*in*_/*K*_*out*_ can help us to distinguish popular(where *K*_*in*_/*K*_*out*_ > 1) and unpopular(*K*_*in*_/*K*_*out*_ ≈ 1) users [34].

**Fig 1:**
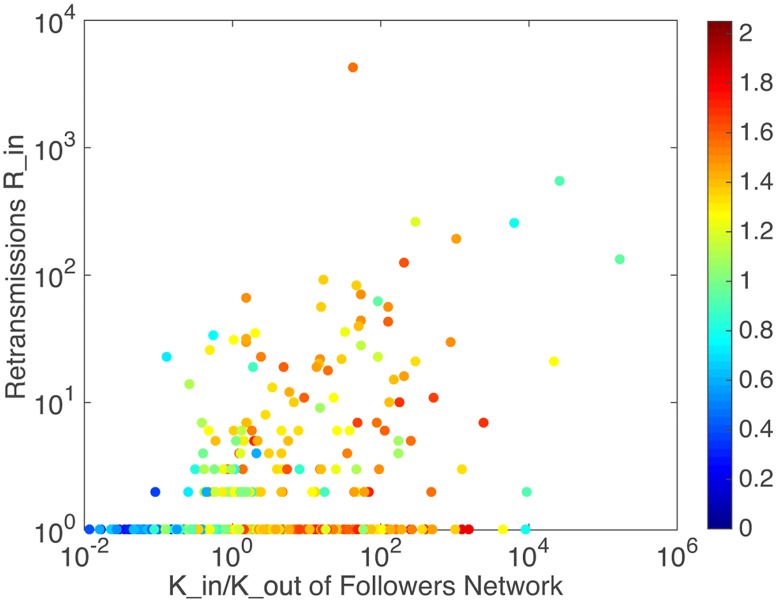
The relationship between retransmissions and users in degree and out degree, colored by user activity.

As mentioned above, a user can retransmit messages from others and be retransmitted by others in Twitter. Therefore, *R*_*in*_ is defined as the amount of retransmissions by others for a user. A greater *R*_*in*_ means a large number of others influenced by the user. It is calculated from the dataset to indicate the ability of a user to spread information to public. Fig 1 shows the relationship between the amount of retransmissions *R*_*in*_ and *K*_*in*_/*K*_*out*_, colored by user activity [42]. User activity is an evaluation indicator of online human behavior defined as the total number of the generated messages. Recent studies found that the activity performed by users on Twitter brought important information to understand a wide variety of phenomenon in society or communication [43, 44].

Roughly, the popular users can be separated from the unpopular users. It can be seen that popular users can gain large amount of retransmissions with low level of activity. Meanwhile, passive ordinary users often pay much more activity to gain the similar amount of retransmissions. Some users without considerable subscribers may get even much more retransmissions than popular users, though they do not perform more activity. In this point of view, the influence a user gains is not necessarily associated with the level of activity that he performed.

**Fig 2:**
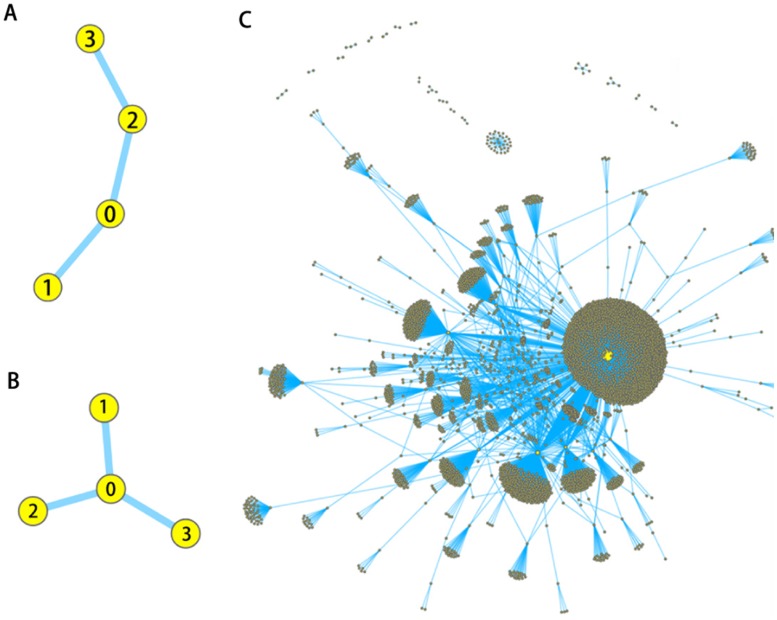
The topological structure of *retweets* *network*. A) Node 1 to 3 retweeted the message that originally posted by node 0 through the green path. Node 3 participated the process after he saw the retweet message from node 2. B) Readers on Twitter could only find the original message from node 0 with no information about the work of node 2 in this process. C) The topology of retweets network can be seen as the aggregate structure of independent star coupled network cascades.

According to retransmission mechanism of Twitter, all retweet messages are merely retransmit the original tweet message, no matter how many users have participated in this process before, no matter who is the reciprocal one of retransmission queue. As shown in part A of Fig 2, for example, Node 1–3 retweet the message posted originally by Node 0 through the green path, Node 3 participate the process in reality after he/she sees the retweet message from Node 2 then click the “retweet” button under this message. However, readers on Twitter can only find the original message from Node 0, without information about the influence of Node 2 in this spreading process, as shown in part B of Fig 2. Those users, who participate in the messages spreading process, play a role as middleware, are difficult to be distinguished from the surface of Twitter.

Since the users’ retransmission behavior on Twitter is actually the retransmission of original tweet messages, for information spreading, the influence of cascades evolution or propagation path greatly reduces, while the influence of essential value of messages, and related user behavior, increases substantially. Actually, many recent studies were focused on how many hopcount a user was away from the source node. However, the efforts of each user to gain influence and get their information spread on the network is a subject that has not yet been explained [42]. As a result, the topology of *retweets* *network* is an aggregate structure of independent star coupled network cascades [45, 46], as shown in part C of Fig 2.

These are critical and practical issues derived from the retransmission mechanism of Twitter. In the sense, it is important to measure how users exert their influence and make their messages spreading among the Twitter network from new perspective. Also, this indicator just reflects the quantitative characteristic of human behavior, but studies suggest that the behavior and interaction of individuals to gain influence may not be measured or explained only from quantitative perspective [47]. Thus our study focused on other statistical features of human behavior that represent the ability of individuals to interact with others and disseminate information.

### Users’ participation

As noted already, even though the users who can hardly be distinguished from the surface of Twitter still play a role as middleware during retransmission, implies that each user has an individual influence to get messages spreading. Specifically, as users can read and retweet any messages by hashtag without the necessity of following others, this user influence may be associated with the time of participation. We searched online user behavior from temporal perspective, and then proposed *user* *enthusiasm*, *user* *engine* and *user* *duration*.

In Twitter network, a message may be retweeted several times at different moment since it was posted. This message spreading process often persists for a period of time. Therefore, *user* *enthusiasm*, *η* can be defined as the average ratio of the time that a user spent responding to a message to the persisting time of the message spreading process. The expression of *user* *enthusiasm* is Eq (1):

$$\begin{array}{c} \eta_{i}=\frac{1}{K}\sum_{k=1}^{K}\frac{t_{k,i}}{t_{k}}, \end{array}$$ (1)

where *t*_*k*, *i*_ is the duration from start of message cascade *k* to the moment that user *i* posted or retweeted the message *k*, *t*_*k*_ is the duration of the message cascade *k* that message *k* involved in, and *K* is the amount of messages posted or retweeted by user *i*. We calculated the time value with equal milliseconds since the Unix Epoch (Jan 1 1970 12AM UTC) and made normalized process to get them more scientific. As individual message *k* is a part of the message cascade *k*, it could be original post or retweet. If message *k* is the original post, it is the start of its cascade, *η*_*i*_ is 0, and in other cases *η*_*i*_ = 0–1. A user’s *η* closer to 0 implies more rapid the user participate in message cascades generally.

However, if we only consider the time value of users’ action, the different scaling properties between intra-day and inter-day activities [17, 48, 49] will take effect. On the other hand, all users that have retweeted a message aggregate into a queue sorted by the moment that they took action. Higher ranking in the queue leads to greater possibility for a user to influence others.

Furthermore, as users are located in different time zones in the world, the direct calculation of time value may not reflect users’ participation accurately. Thus *user* *engine*, *β*, is defined as the average ranking status of a user in each message spreading process that he/she participates in. The expression of *user* *engine* is Eq (2):

$$\begin{array}{c} \beta_{i}=\frac{1}{K}\sum_{k=1}^{K}\frac{s_{k,i}}{s_{k}}, \end{array}$$ (2)

where *s*_*k*, *i*_ is the amount of users sorted behind user *i* in message cascade *k*, *s*_*k*_ is the total amount of users that participate in message cascade *k*, and *K* is the amount of messages posted or retweeted by user *i*.

Meanwhile, to get more analysis about users participation, the duration of users’ activities, including posting and retweetting, is studied. The *user* *duration*, *μ*, is defined as the average duration of a user in each message spreading process that he/she participates in. It reflects the sustainability of users’ behavior. The expression of user duration is Eq (3):

$$\begin{array}{c} \mu_{i}=\frac{d_{i}}{d_{tot}}, \end{array}$$ (3)

where *d*_*i*_ is the duration of activities of the user *i* in the whole event, *d*_*tot*_ is the total duration of this discussion on Twitter.

The user activity represents how many actions that each user performed, the user enthusiasm and engine reflect how proactive or energetic each user is during each action, while the user duration implies the sustainability of users’ action. They are all statistical indicators of users’ action at different dimensions. Empirical datasets were used to calculate and find how these indicators are associated with the efforts of users on Twitter to get their information spreading.

### Community analysis

To further analyze the users’ behavior and influence, the community structure [50, 51] is calculated for *retweets* *network*, which is a more appropriate structure where information actually spreading through [34]. The communities in the *retweets* *network* is gained based on the Louvain algorithm [52], which is of good computing performance on the modularity optimization for direct network.

Then, the users’ influence of large communities, and the collectivity, are studied.

## Results

### Retweets network and followers network

We considered messages posted on Twitter as a case study, which consisted of posting messages identified with the hashtag #AFCvMCFC, talking about the event that Arsenal FC against Manchester City FC in the Premier League [53]. All the original tweets and retweets included the hashtag #AFCvMCFC on December 14–24, 2015. Each data contained information about its message ID, user ID, user profile, created time and message content. In total we found 417,064 messages, written by 32,375 users.

The *retweets* *network* and *followers* *network* were built, with the methods we mentioned in Section 2, to analyze the users’ roles in the information spreading dynamic during the discussion, as shown in Table 1.

**Table 1:** Retweets network and followers network.

Networks	Nodes	Edges	Mean distance
*Followers* *Network*	9782	221,498	2.53
*Retweets* *Network*	7747	53,454	3.08

### Empirical results of users participation

**Fig 3:**
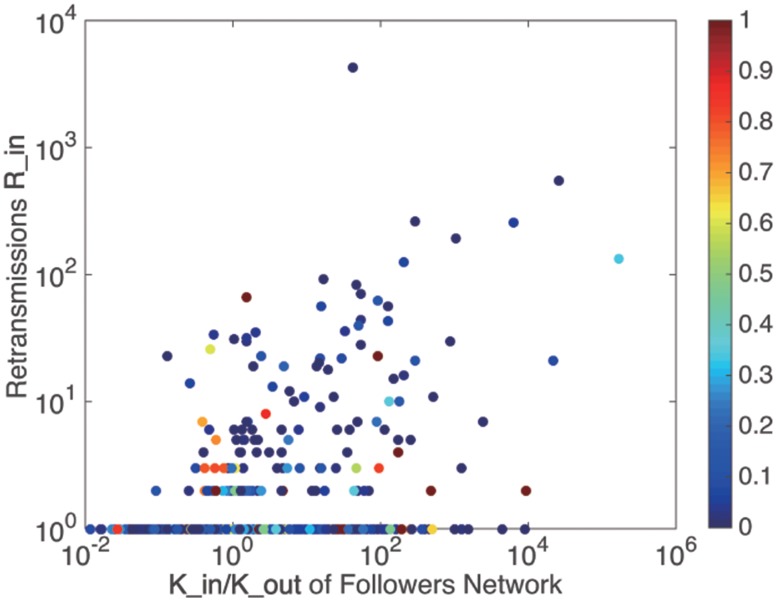
The relationship between retransmissions and users in degree and out degree, colored by user enthusiasm.

**Fig 4:**
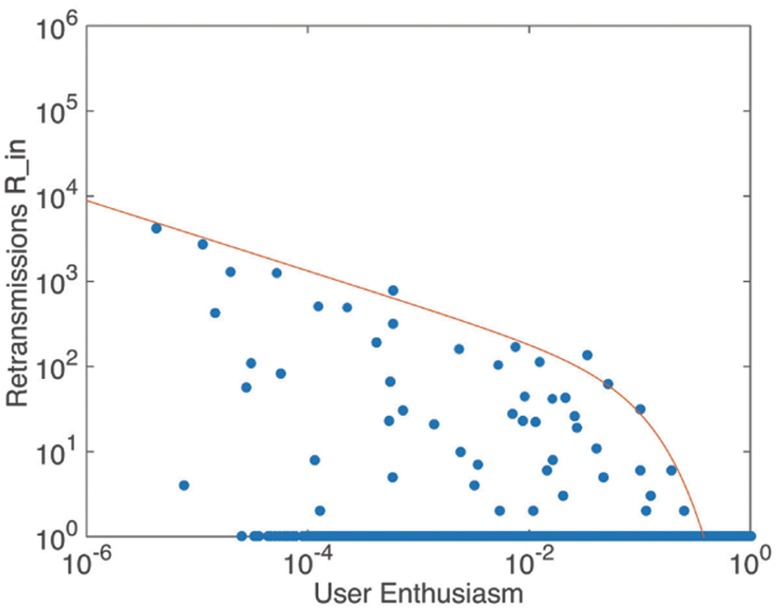
The relationship between retransmissions and user enthusiasm. The solid envelope line follows power function, *y* = *ax*^ − *b*^ *e*^ − *x*/*c*^, where *a* = 29.5 ± 0.81, *b* = 0.42 ± 0.023, *c* = 10.

Fig 3 shows the relationship between the retransmissions *R*_*in*_ and *K*_*in*_ /*K*_*out*_. The colored plots represent the *user* *enthusiasm* *η*. Fig 4 shows the relationship between user enthusiasm and the amount of in retransmissions. It can be seen that almost all the users who gained retweets more than once present a *user* *enthusiasm* of *η* = 0–0.3(colored by blue). Meanwhile, some users who achieve this condition may not be able to get large amount of retransmissions, i.e. a *user* *enthusiasm* of 0–0.3 is a necessary but not sufficient condition for large amount of retransmissions. The envelope in Fig 4 can be well fitted by the power function in Table 2.

**Table 2:** Envelope fitting results of the relationship between retransmission(y) and measures(x).

Measures	Functions
*Userenthusiasm*	*y* = *ax*^ − *b*^*e*^ − *x*/*c*^, *a* = 29.5 ± 0.81, *b* = 0.42 ± 0.023, *c* = 10
*Userengine*	*y* = *ax*^*b*^, *a* = 2308.4 ± 2.6, *b* = 5.79 ± 0.012
*Userduration*	*y* = *ax*^*b*^*e*^*x*/*c*^, *a* = 4112.7 ± 4.9, *b* = 1.02 ± 0.025, *c* = 1000

**Fig 5:**
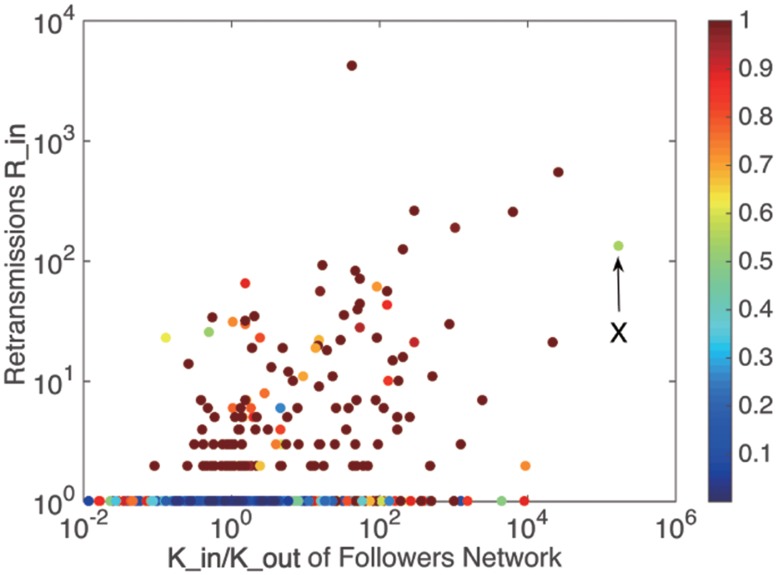
The relationship between retransmissions and users in degree and out degree, colored by user engine.

**Fig 6:**
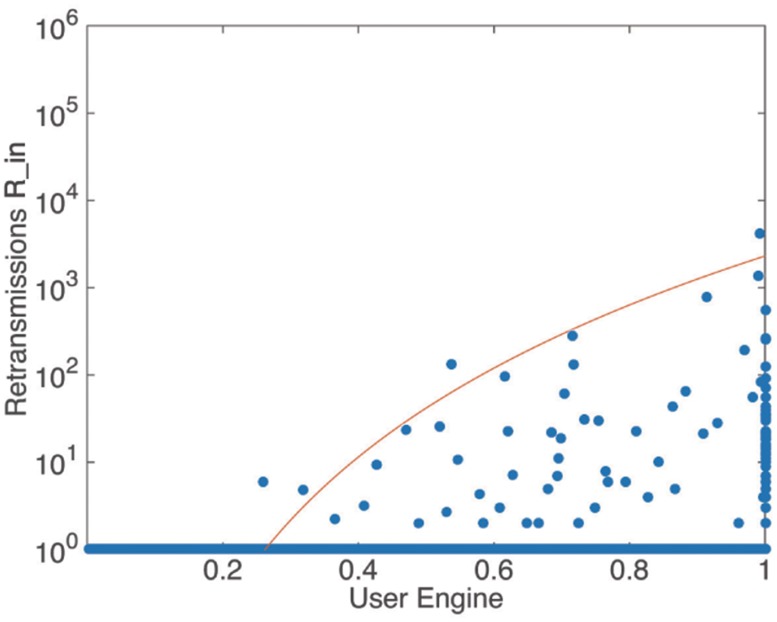
The relationship between retransmissions and user engine. The solid envelope line follows power law distribution, *y* = *ax*^*b*^, where *a* = 2308.4 ± 2.6 and *b* = 5.79 ± 0.012.

If a user posts a message originally, this message may be read by all the others in the cascade, i.e. *β*_*i*_ = 1, and in other cases *β*_*i*_ = 0–1. A closer-to-1 *β* implies more chances the user has to affect others in each cascade. Figs 5 and 6 show the relationship between the retransmission and *user* *engine*. It can be seen that almost all the users retweeting more than once has a large *user* *activity* *β* (dark red in Fig 5).

We also fitted the envelope of these scatters and found that they follow power law distribution, as shown in Table 2. The *user* *enthusiasm* and *user* *engine* are strongly negatively correlated to each other. Actually, *user* *enthusiasm* is defined as the average ratio of the time that a user spent responding to a message to the persisting time of the message spreading process. The *user* *engine* is defined as the average ranking status of a user in each message spreading process that he/she participates in. To summarize, *user* *enthusiasm* measures the action with the actual time value, while *user* *engine* measures users’ action with the order of message spreading queue. *User* *engine* can avoid the impact from intra-day and inter-day activities and the problem of different time zones, and it seems like a better indicator in this perspective. However, the actual time value is practical datum generated from users and can reflect real situation of individual’s participation [42]. Thus *user* *enthusiasm* is also an important indicator to be considered.

**Fig 7:**
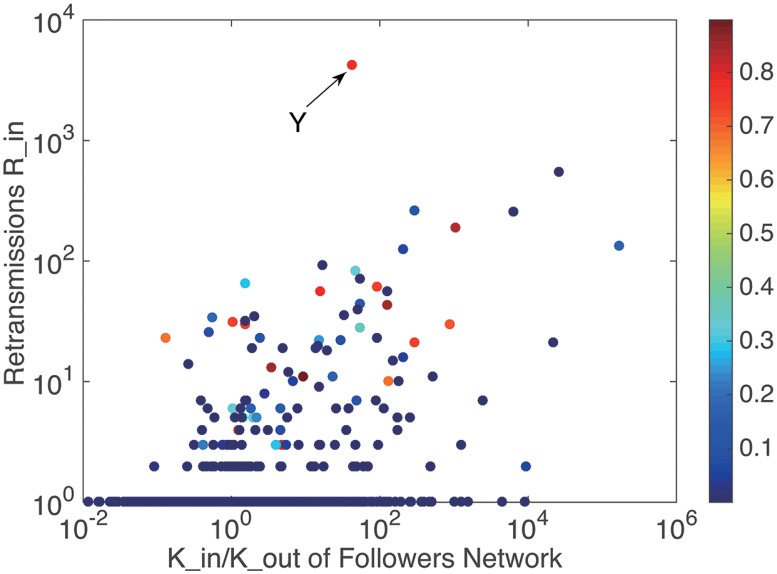
The relationship between retransmissions and users in degree and out degree, colored by user duration.

**Fig 8:**
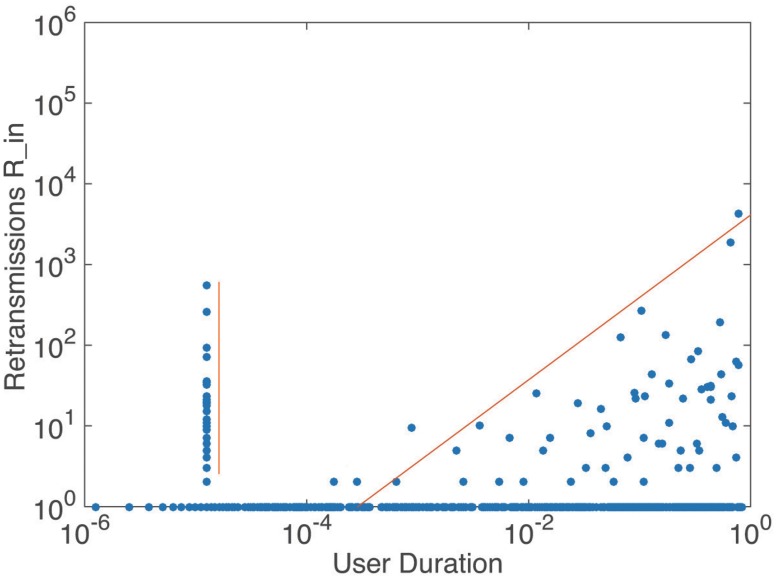
The relationship between retransmissions and user duration. The scatters on the left follow a linear distribution. The envelope of the right part follows power law distribution, *y* = *ax*^*b*^ *e*^*x*/*c*^, where *a* = 4112.7 ± 4.9, *b* = 1.02 ± 0.025, *c* = 1000.

We presented the status of user duration in Figs 7 and 8. It can be seen that most users present a small *user* *duration* *μ* (blue colored in Fig 7). That means most of the ordinary users participate in the discussion in a very short period of time, but they may gain no retransmission and large number of retransmission. Besides, there are still some users keep posting or retweetting all the time or in a long period of time, and they all get considerable amount of retransmission. The analytic expression of the envelope in Fig 8 is a piecewise function, where most scatters follow linear distribution with small *user* *duration* and the others follow power law distribution with large *user* *duration*, as shown in Table 2.

Based on an overall consideration of the results, it can be found that users who can gain considerable amount of retransmission mostly present large value of *user* *engine*, but small values of *user* *enthusiasm* and *user* *duration*. However, there are some special users, like user *X* in Fig 5 with a small *user* *activity* but a large *user* *duration* and user *Y* in Fig 7 with a very large *user* *duration*, can gain large amount of retransmission in other ways.

In addition, as mentioned in Section 2, we randomly selected a user as the missing user and introduced some random factors in empirical data. This means that the data points below the envelope curves are almost homogeneous. We considered another strategy of data preprocessing by removing the incomplete data, but the power-functional-like envelope curves could still be seen, and the homogeneous data points decreased. The results are shown in Supporting Information.

This results indicate that our method of data preprocessing has not influenced the correctness of analytical conclusion. However, removing the incomplete data will cause the loss of information of user profile and user action. It is a complex subject to balance the data accuracy and completeness, and actually, it is also what we want to do in our further study. We will try to get more complete data and find more scientific methods in our further research.

### Community structure

**Fig 9:**
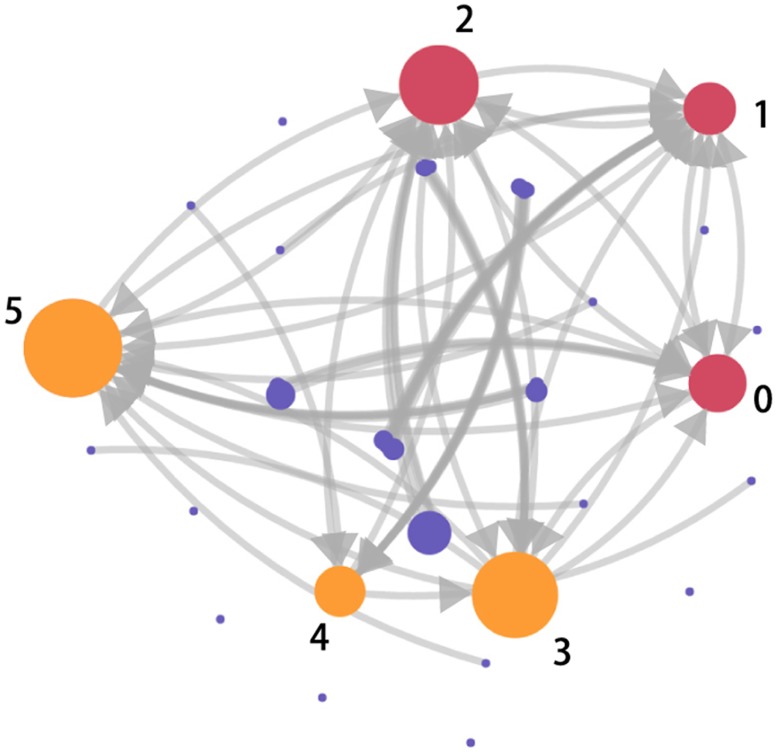
Community structure for the retweets network. Large communities are generated around sport app account (0), Arsenal FC’s official charity (1), Arsenal FC’s official account (2), Arsenal FC’s superstar (3, 4) and chairman of fans association (5). Large communities can be categorized as information creators (red circles) and information promoters (yellow circles), while small communities (purple circles) widely distribute in the network.

In the *retweets* *network*, six large communities and 26 small communities were generated by users of similar collectivities. The community structure for the *retweets* *network* is shown in Fig 9. The nodes in different size represent communities that consist of different amount of users. The edges with different width represent different amount of direct links between communities.

**Fig 10:**
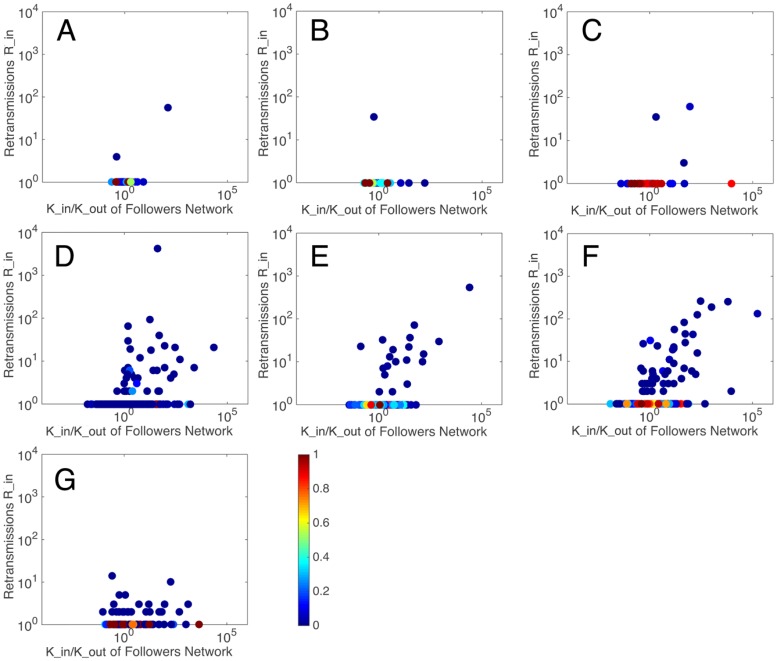
The relationship between retransmissions and users’ in degree and out degree (colored by user engine) among seven collectivities. Sport app account (A), Arsenal FC’s official charity (B), Arsenal FC’s official account (C), chairman of fans association (D), Arsenal FC’s superstar (E, F) and small communities(G).

**Fig 11:**
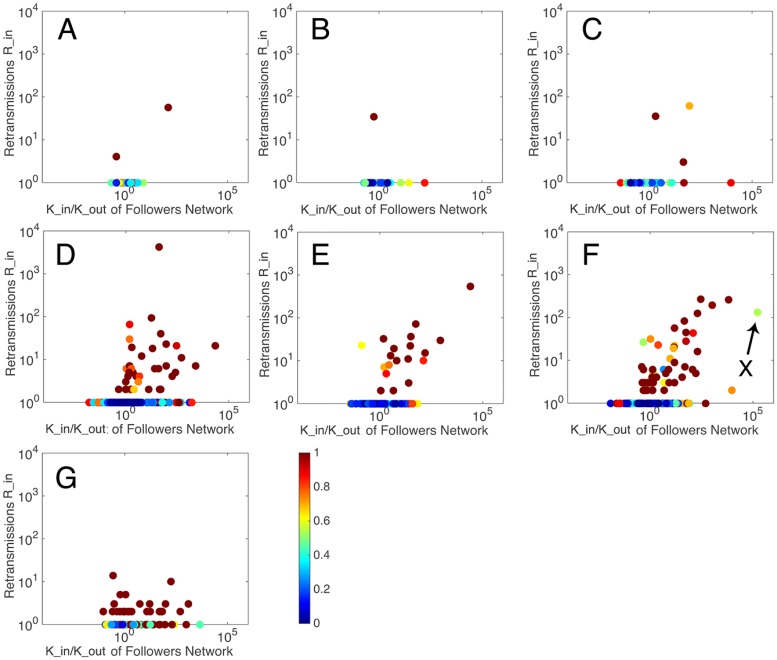
The relationship between retransmissions and users’ in degree and out degree (colored by user enthusiasm) among seven collectivities. Sport app account (A), Arsenal FC’s official charity (B), Arsenal FC’s official account (C), chairman of fans association (D), Arsenal FC’s superstar (E, F) and small communities(G).

**Fig 12:**
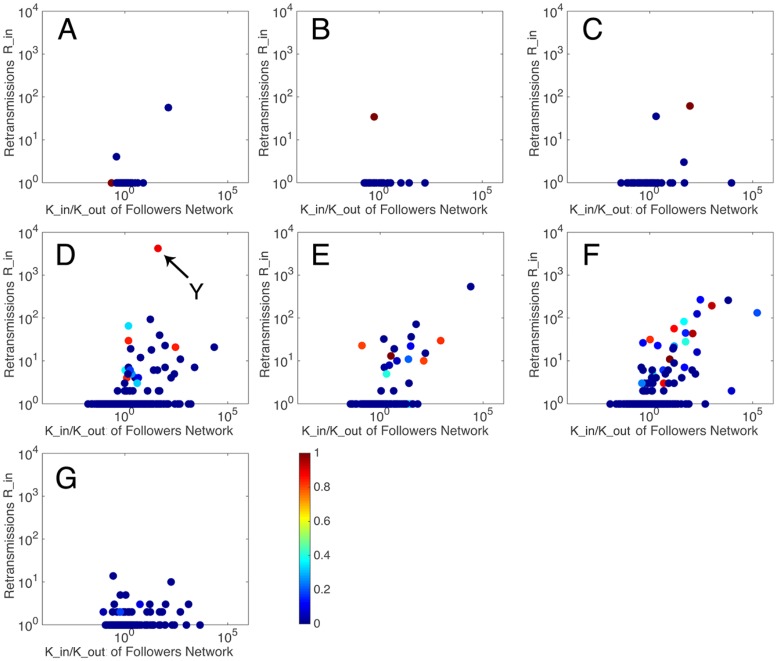
The relationship between retransmissions and users’ in degree and out degree (colored by user duration) among seven collectivities. Sport app account (A), Arsenal FC’s official charity (B), Arsenal FC’s official account (C), chairman of fans association (D), Arsenal FC’s superstar (E, F) and small communities(G).

The small communities have less than 10 users, while large communities have over 1000 users. The users in each community always perform around at least one central user. The large communities are generated around sport app account (0), Arsenal FC’s official charity (1), Arsenal FC’s official account (2), Arsenal FC’s superstar (3, 4) and chairman of fans association (5). The large communities are highly retransmitted than small communities, while small communities, which distribute widely in the network, are regarded as a collectivity. Then, the users’ influence of large communities and the collectivity are studied. Figs 10, 11 and 12 show the performance of *user* *engine*, *user* *enthusiasm* and *user* *duration* in these communities, respectively.

It can be seen that there are three types of scatter distribution among the seven collectivities. In Type 1 distribution, few central users appear with many ordinary users who are retransmitted once or less, and the amounts of retransmissions of the central users are also not large. This occurs in the sport app account community, Arsenal FC’s official charity community and Arsenal FC’s official account community. In Type 2 distribution, central users appear with large amounts of retransmissions, while users around them also gain certain amounts of retransmissions. This occurs in Arsenal FC’s superstar community and chairman of fans association community. In Type 3 distribution, most users gain small amounts of retransmissions, without obvious central users. This occurs in the collectivity of small communities.

Based on the results, the users can be divided into four categories: information creators, information promoters, information supporters and information consumers. Information creators are the central users in Type 1 distribution. They belong to the professional or non-professional mass media accounts related to the subject of this event, like non-professional sports media related to football and professional Arsenal FC’s media related to the match. They tend to follow lots of accounts, especially popular accounts, while they have much more amounts of followers. Each of them presents a large value of *user* *engine* and small values of *user* *enthusiasm* and *user* *duration*. Then, they can gain certain amount of retransmission and generate some information cascades. However, the other users in these cascades can hardly gain retransmission. That means information creators can only create and slightly spread information, but cannot make others participate in activities, because they are too official or formal for people to interact with. Their influences are limited, and their audiences receive and retweet information passively. The overall status of their values of *user* *enthusiasm* shows that information creators are the sponsors of information spreading with their limited influences.

Information promoters are the central users in Type 2 distribution. They belong to famous or vital individuals related to this event, like superstars and representatives of supporters. As we mentioned before, there are some special users having great influence with abnormal *user* *engine* or *user* *duration*. They are all information promoters. For example, user *X* in part F of Fig 11 is a superstar, Mesut Özil, who participated in this discuss not so rapidly but influenced thousands of people, user *Y* in part D of Fig 12 is a representative of fans, Morgan Rubes, who posted and interacted with others continuously. They cannot only gain extremely large amount of followers and retransmission, but also drive people around them to discuss, create information and get retransmission, which implies that information promoters have ability to create information and affect others to create information. They are the critical section in each information cascade to determine the depth of information and their influences are special and great.

Information supporters are the central users in Type 3 distribution. They can be considered as ordinary users who just have slightly strong influence to make their friends get together to discuss, like people who are active and popular or make friends widely in daily routine. Their values of *user* *enthusiasm* and *user* *duration* are small, but their values of *user* *engine* are large as they usually focus on the event all the time, hence the small but widely-distributed communities in the whole social network. The topics in these communities vary independently, producing large amount of varied cascades of different sizes. That means information supporters determine the width of information in the information spreading.

Information consumers are the ordinary users who consume and retweet messages but hardly create anything. They widely appear in each community, playing a role as passive audiences. They do not participate in the information spreading proactively, but they are the largest group of users in the network.

## Conclusions

In this paper, we discussed the special mechanisms for information propagation on Twitter, as the existing topological measurements that based on propagation path could not explain the efforts of individuals to get their information spreading accurately and reasonably. We found that most users played a role as middleware during the information spreading process. Thus we proposed a measurement with three measures, *user* *engine*, *user* *enthusiasm* and *user* *duration*, to state the relationship between their participation and the influence they gained. We also conducted a community analysis to study the structure and dynamic of information spreading network. We found that users were organized into four different categories: information creators, information promoters, information supporters and information consumers, presenting different participation and different influence on others. We concluded that users who can gain large amount of retransmissions presented a large value of *user* *engine* and small values of *user* *enthusiasm* and *user* *duration*. And finally we analyzed the information spreading process in the four categories based on this pattern. However, at current stage, the given conclusions we gave are qualitative and analytical for this subject. So, it is meaningful to find finer quantitative conclusion in the further study. Our current study can help for deeper insight in online social dynamic and information spreading.

## Supporting information

S1 Fig The relationship between retransmissions and user enthusiasm without random and incomplete data. The solid envelope line follows power function, *y* = *ax*^ − *b*^ *e*^ − *x*/*c*^, where *a* = 29.5 ± 0.81, *b* = 0.42 ± 0.023, *c* = 10.
(EPS)

S2 Fig The relationship between retransmissions and user engine without random and incomplete data. The solid envelope line follows power law distribution, *y* = *ax*^*b*^, where *a* = 2308.4 ± 2.6 and *b* = 5.79 ± 0.012.
(EPS)
